# Randomized trial of transcutaneous auricular vagus nerve stimulation on patients with disorders of consciousness: A study protocol

**DOI:** 10.3389/fneur.2023.1116115

**Published:** 2023-04-13

**Authors:** Lijuan Cheng, Lingxiu Sun, Lu Xu, Falin Zhao, Xiaochen Liu, Anqi Wang, Haibo Di, Yu-Sheng Cong

**Affiliations:** ^1^Key Laboratory of Aging and Cancer Biology of Zhejiang Province, School of Basic Medical Sciences, Hangzhou Normal University, Hangzhou, China; ^2^Qianjiang College, Hangzhou Normal University, Hangzhou, China; ^3^International Unresponsive Wakefulness Syndrome and Consciousness Science Institute, School of Basic Medical Sciences, Hangzhou Normal University, Hangzhou, China; ^4^School of Public Health, Hangzhou Normal University, Hangzhou, China

**Keywords:** transcutaneous auricular vagus nerve stimulation, disorders of consciousness, acquired brain injury, randomized controlled trial, vagal cortical pathways model, multi-modal assessment

## Abstract

**Background:**

Transcutaneous auricular vagus nerve stimulation (taVNS) has recently been explored for the treatment of Disorders of consciousness (DoC) caused by traumatic brain injury. The evidence of taVNS during the consciousness recovery has been recently reported. However, the mechanism of taVNS in the recovery of consciousness is not clear. This study attempts to investigate the effectiveness of taVNS in DoC by means of Coma Recovery Scale-Revised (CRS-R), Magnetic resonance imaging (MRI), Electrophysiology (EEG), and Single-molecular array (Simoa).

**Methods/design:**

Nighty patients with DoC acquired brain injury are randomized into one of three groups receiving sham taVNS or active taVNS (just left and left or right), respectively. Each of the three groups will experience a 40 days cycle (every 10 days for a small period, baseline 2 weeks, intervention 2 weeks, 40 min per day, 5 days per week, then no intervention for 2 weeks, intervention 2 weeks, 40 min per day, and 5 days per week). Primary outcomes (CRS-R) will be recorded five times during every period. Secondary outcomes will be recorded at the first and at the last period [MRI, EEG, Phosphorylated tau (P-tau), and Neurofilament light chain (NFL)]. We will take notes the adverse events and untoward effects during all cycles.

**Discussion:**

Transcutaneous auricular vagus nerve stimulation as a painless, non-invasive, easily applied, and effective therapy was applied for treatment of patients with depression and epilepsy several decades ago. Recent progress showed that taVNS has behavioral effects in the consciousness recovery. However, there is no clinical evidence to support the effects of taVNS on brain activity. Therefore, we will design a randomized controlled trial to evaluate the effectiveness and safety of taVNS therapy for DoC, and explore neural anatomy correlated to taVNS during the consciousness recovery. Finally, this protocol also tests some biomarkers along with the recovery of consciousness.

**Clinical Trial Registration:**

Chinese Clinical Trial Registry, ChiCTR2100045161. Registered on 9 April 2021.

## 1. Introduction

Acquired brain injury is often known as a “silent epidemic disease,” which is a general term for all brain injuries (1, 2). Acquired brain injury includes infection, hypoxia, and trauma, which can cause systemic or focal injury, and a series of pathophysiological changes (such as brain hematoma, brain contusion, and diffuse axonal injury). There are about 1.7 million Traumatic brain injury (TBI) events occur every year in the United States (3). But in China, there are no accurate epidemiological investigation data. The survival rate of Disorders of consciousness (DoC) patients after severe brain injury has been significantly increased due to advanced medical treatment and healthcare (4). However, it caused the heavy economic burden and great mental stress for family members. Currently, early assessment and health management of DoC are facing a great challenge because of its unclear pathophysiological nature. Understanding the biological basis of DoC would have important implication for the development of diagnostic and therapeutic strategies.

Dysfunction of the brain in certain areas, combined with the disruption of the neural connections between and within neural networks, can lead to DoC (4). DoC patients are in coma kept closed eyes, lack of arousal, and awareness (5), and do not respond to environment. Unresponsive wakefulness syndrome (UWS) of DoC (previously called Vegetative State, *VS*) is defined by some intermittent spontaneous arousal observed after tactile, auditory, or painful stimulation (1), but these DoC patients in no self-consciousness cannot consciously interact with their surrounding environment (6). Minimally consciousness state (MCS) patients have arousal and the ability to consciously interact with the environment, such as visual tracking, object localization; reaching, or instruction following (6). These changes are unstable but reproducible. MCS can be further divided into minus (MCS−) or plus (MCS+) (7). While consciousness is fluctuating in MCS, it is still possible to have the residual consciousness behavior can be monitored by careful assessment.

To date, there is no single effective way to predict, evaluate, and intervene in early severe brain injury (2), Therefore, exploring accurate assessment methods and effective intervention strategies for DoC are urgently required in this emerging fields. Transcutaneous auricular vagus nerve stimulation (taVNS) is a potential intervention in treatment of DoC. Certain visceral representative areas of the ear region produce brainstem-to-central neural modulation effects similar to invasive vagus and neural stimulation (8, 9). Because taVNS is non-invasive and has no need for surgical condition (8, 9), low cost and easy to perform for DoC patients in families or communities. It has been shown that taVNS can produce a beneficial effect in DoC (4), but the underlying mechanism is unclear.

The auricular branch of the vagus nerve is the source of sensory information to the ear. The external ear’s central area contains sensory fibers, which are situated close to the Cymba Concha section of the auricle. The auricular vagus nerve’s afferent neurons go to the inferior vagal ganglion, and then the impulse is sent to the solitary tract nucleus in the brainstem. The solitary tract is the primary receptor of sensory information from different branches of the vagus nerve, which then sends out various signals to other parts of the body, such as the locus coeruleus. This area of the reticular activating system is a major source of adrenaline-inducing projections that reach the cortex, subcortex, and brainstem. It appears that the activation of the locus coeruleus may be the cause of many of the observed therapeutic outcomes from VNS and taVNS (10). The firing of neurons in the locus coeruleus causes a massive release of norepinephrine in the thalamus and hippocampus, which is an essential part of the noradrenergic pathway and is crucial for alertness, arousal, and the fight-or-flight reaction (11). The vagus nerve in humans stimulates metabolic processes in the forebrain, thalamus, and reticular formation, and it also regulates activity in the brainstem, as well as the nucleus of the solitary tract (NTS), dorsal raphe nuclei, amygdala, and hippocampus (11).

Based on our current understanding of taVNS related to the awareness-related neural pathways, vagal cortical pathway model has been proposed (4). The vagal cortical pathway model through six distinct strategies to influence brain activity and consciousness: boosting the excitability of the ascending reticular activating system, activating the thalamus, reconstructing the cortical striatal-thalamic-cortical network, establishing negative connections between external and default mode networks by stimulating salience networks, stimulating the norepinephrine pathway to raise external network activity and connectivity, and raising excitability within the default mode network through the serotonin pathway (4). This model was constructed to investigate the mechanism of action that taVNS exerts neural modulatory effects as therapeutic intervention in the management of DoC. Therefore, the vagal cortical pathway model should be demonstrated in prospective randomized controlled clinical trials. This current study, with its randomly chosen participants, seeks to (1) gauge the effectiveness of taVNS in DoC; (2) confirm or refute vagal cortical pathway model (10, 12, 13); and (3) identify biomarkers for diagnosis and targets for therapeutic intervention (2).

## 2. Methods

### 2.1. Study design

**Figure 1:**
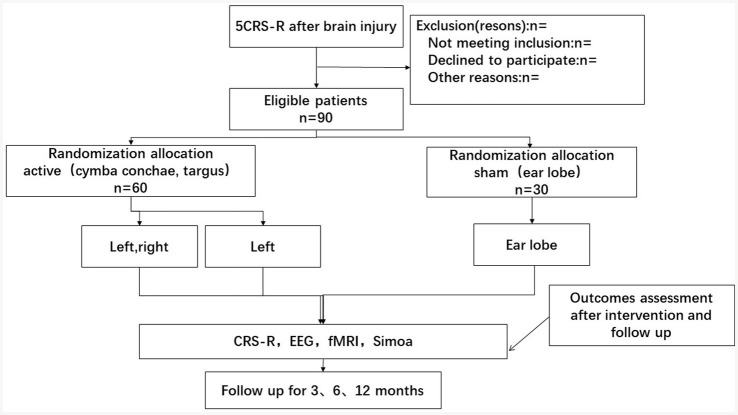
Flowchart of the study.

Ninety patients suffering from DoC will be chosen for this prospective, randomized trial which involves double-blinded assessments. The participants will be randomly divided into the intervention and control groups with an even distribution. The control group will be administered ear lobe therapy, in contrast to the intervention group which will receive the active taVNS. We will stimulate left cymba conchae for one group, the second group subjects will be stimulated left cymba 20 min and right cymba 20 min once a day. Because we have not seen the side effects from the clinical practice, and the right vagus nerves modulate the left hemisphere. This study will last 40 days and a half-year observation period will follow. CRS-R behavioral improvement will be monitored five times every 10 days. At the beginning and the end of the fourth period, the following tests will be carried out: EEG, MRI, and measurement of certain biomarkers related to brain injuries. All side effects during each treatment will be documented. This study will be implemented in Shanghai Yongci Hospital, and rehabilitation therapists are held accountable for following the standard operating procedure and evaluating the trial’s progress at all clinical sites. This trial is illustrated in Figure 1.

### 2.2. Participants and recruitment

Participants will be recruited from the neurologic care unit. The study will be advertised at the bedside between April 2021 and December 2022. Researchers will select potential candidates by reviewing their medical records in the Computerized Patient Record System (CPRS) and communicate the details of this trial to their families. Following the agreement of the participants’ guardians, a neurologist will review them based on the eligibility criteria. Those who meet the criteria have been invited to join the experiment and must go through a neurological examination before being evaluated for rehabilitation. All study participants must give permission by their family members.

Patients who meet the following criteria will be deemed eligible for the trial: (1) age > 18 years, onset >28 days; (2) patients were assessed by using CRS-R (five times) as DoC (UWS, MCS−, and MCS+); (3) patients had not received central stimulants drugs within 48 h before enrollment; (4) no neuromuscular blockers were applied within 24 h prior to enrollment; and (5) no facial or ear pain, no recent ear-related injuries, no metallic prosthetics, and no pregnancy. Patients who meet the following criteria will be excluded from the trial: (1) personal or familial history of seizures and history of cardiovascular disease, with developmental neurological or psychiatric illness history; (2) untreated brain edema; (3) metal implants in the body; (4) unstable vital signs; and (5) contraindications: such as claustrophobia.

### 2.3. Sample size

The main outcome index of this study is the CRS-R scale score, which is set as a paired experiment count, the sample estimation formula is as follow:

$$n=\frac{{\left(Z_{\alpha /2}+Z_{\beta }\right)}^{2*}\sigma^{2}}{\delta^{2}}$$

According to the results of previous studies (4), the estimated difference standard deviation is 15, and the difference is 10, set two-sided = 0.05, and the grasp degree is 90%. According to the sample size calculation formula, 24 subjects were needed. Considering the loss of visits and refusal, at least 30 subjects were needed. Therefore, 30 *VS*/UWS and MCS patients will be randomly selected for each sample group, with a total sample size of 90.

### 2.4. Randomization

The study will utilize block randomization. The participants will be classified into either the “MCS group” or the “UWS/*VS* group.” All subjects will be randomly divided into the intervention group and control group in a 1:1:1 ratio. To ensure impartiality, a random number will be used to produce a random allocation order, this task will be handled by a person who is not involved in the trial. The order of random allocation will remain undisclosed to those assessing the outcomes and the statistician. After evaluating the baseline information of the suitable participants, their healthcare personals and therapists (including acupuncturists and cognitive therapists) will not know patients random number assigned.

### 2.5. Trail protocol

**Figure 2:**
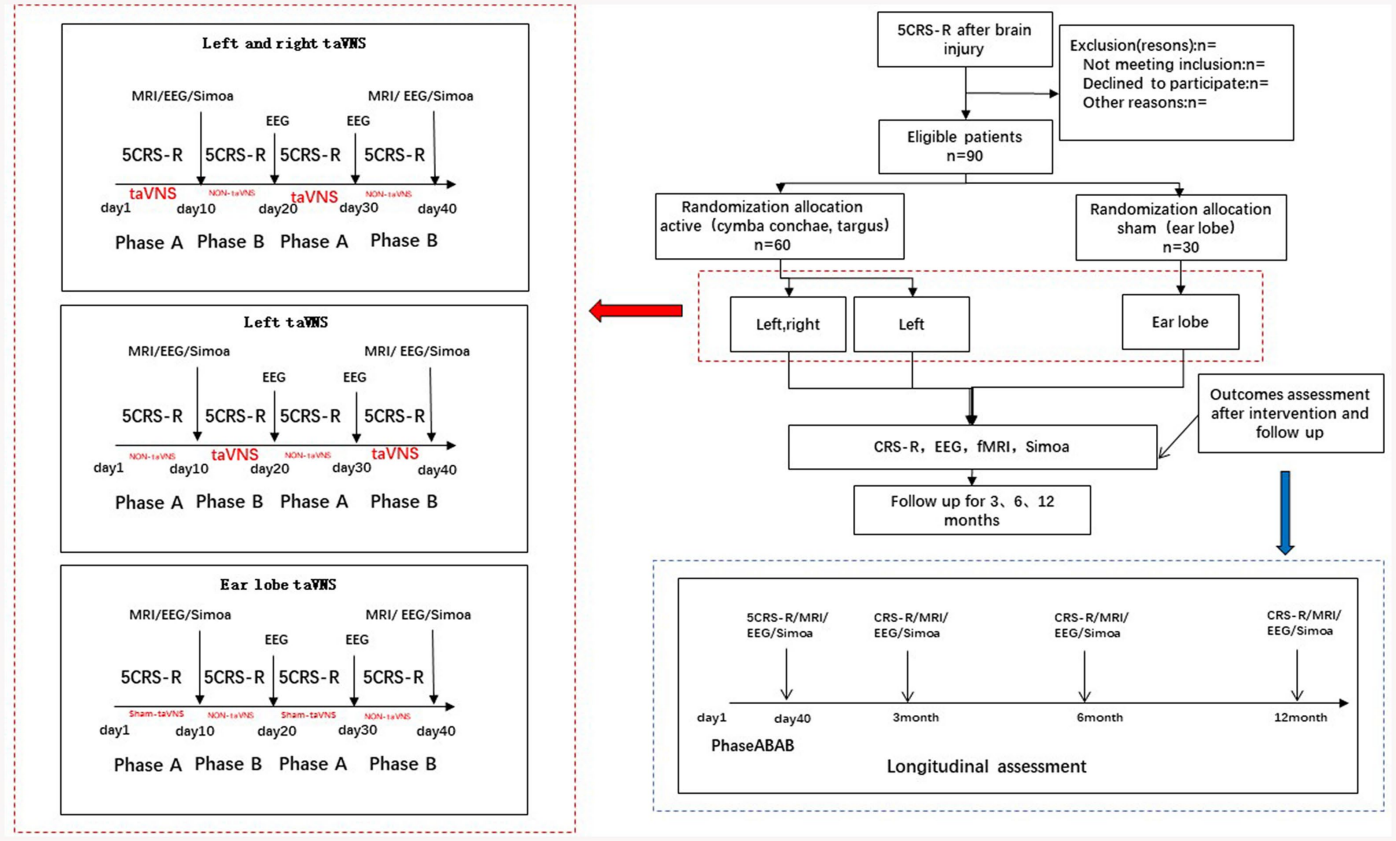
The taVNS stimulation protocol.

Ninety patients will undergo two sessions 10 days apart, with CRS-R and EEG performed before and after VNS. The active session will stimulate the left or right cymba conchae and inner tragus (TENS-200A, SN number: E200A16A000144, the B mode type; stimulation parameters: output pulse width degree of 200us, 20 Hz output 7 s, 4 Hz output, and 3 s alternate cycle output). The taVNS stimulator will be monitored according to patients’ response. The sham stimulation will be applied over the earlobe. We expect behavioral and EEG metrics to be improved in a majority of patients (See Figure 2).

### 2.6. Follow-up

The clinical trial concluded 3 months ago and since then, behavioral scale data have been gathered at regular intervals of 3, 6, and 12 months. For eligible subjects, the MRI/EEG will be performed, and blood samples will be collected for target protein determination.

### 2.7. Blinding

The trial has been designed to be double-blind; thus, an external party that is unaffiliated with the experiment will monitor and oversee the execution of the blinding protocol, with the random numbers placed in a sealed envelope. Evaluators are not involved in the process of recruiting and managing patients, while other investigators, operators, and statisticians act on their own. Prior to the conclusion of the statistical analysis, unblinding is done to counteract the analyst’s potential subjectivity in data analysis; meantime, the analyst knew that the patients have been split into three groups, but was unaware of which one is the intervention group. Following the statistical study, the second step of unblinding is executed to identify which group is the treatment group.

### 2.8. Ethics issues

The Ethics Committee of Hangzhou Normal University [NO: (20190083)] and Shanghai Yongci Hospital [No: (YCYY-20211021-003) have both given the green light for the study protocol and consent forms, respectively]. We will ensure that we have the approval of family members of subjects before proceeding.

### 2.9. Baseline data

This study will collect descriptive data including gender, age, onset, weight, etiology etc. before randomization. The CRS-R was used to evaluate DoC’s consciousness level, and this will be applied initially to check if the three groups are the same prior to the intervention.

### 2.10. Outcome measures

The primary outcome for this study, coma recovery as measured by the Coma Recovery Scale-Revised (CRS-R), will be assessed at five time points for each period, ranging from the first to the fourth. This data will be used to determine the efficacy of the intervention being studied. The secondary outcomes of this study will be measured at both the baseline and the end of the fourth week of the study. Specifically, EEG, MRI, P-tau, and NFL will be used to measure these outcomes. These measurements will provide valuable insight into the effects of the study and help to further understand the results. Any detrimental outcomes or side effects will be noted during the course of the treatment.

### 2.11. Primary outcomes

#### 2.11.1. Coma recovery scale-revised

The CRS-R is used to measure the progress of individuals suffering from DoC (UWS,MCS−, and MCS+) (7). The CRS-R is made up of 23 components organized into six sections that evaluate auditory, visual, motor, oromotor, communication, and arousal functions. Scores that are higher indicate conscious-related behavior, while lower scores signify reflexive activity. The evaluation of performance is dependent on the occurrence or lack of the specified action in response to standard prompts (14).

### 2.12. Secondary outcomes

#### 2.12.1. Electrophysiology

The benchmark for assessing levels of consciousness is the CRS-R (15, 16), whereas EEG is a crucial diagnostic and prognostic tool for patients with DoC (17). Recent advancements in the field have enabled researchers to develop feature-based measures like spectral power analysis, functional connectivity, and complexity measures, for the purpose of diagnosing and predicting outcomes (18–20). Despite this, their use to assess the efficacy of treatments has not been implemented (More EEG details are shown in Supplementary material). In this study, we would analyze spectral power analysis mostly to identify an improvement on EEG (such as alpha power) (17).

#### 2.12.2. Magnetic resonance imaging

In this study, rest Fmri research must always include a brainstem assessment in order to verify the Vagal Cortical Pathways model’s pathways. This can be confidently relied upon as a guarantee of quality in taVNS studies, as it clearly demonstrates the stimulation of the vagus nerve (4). Furthermore, subcortical regions like the thalamus and striatum should be taken into account. Brain connectivity can be estimated by utilizing the thalamus, frontal lateral cortex, anterior cingulate cortex, insula, and posterior cingulate cortex as seed regions in Fmri studies. Moreover, measuring brain activity during tasks should be the preferred approach (21, 22). In severe TBI, an increase in fractional anisotropy (FA) on the initial postinjury diffusion tensor imaging (DTI) was associated with a favorable outcome, and these results suggest that it is secondary to axonal regrowth during later recovery. A variety of other cognitive and functional outcomes in mild to severe TBI have been correlated to DTI (23). Finally, DTI-FA should be analyzed in our study (More MRI details are shown in Supplementary material).

#### 2.12.3. Single-molecule Array

The peripheral blood will be taken regularly according to the experimental protocol, imaging samples will be selected, and the target protein determination will be performed using Simoa HD-1 (Single-Molecule Array Analyzer), known as the digital ELISA with more than 1,000 times higher sensitivity than conventional ELISA (24).

The enrolled subjects will be collected peripheral venous blood before and after the taVNS intervention. Samples with obvious imaging effects will be selected, and the target protein (p-tau, NFL) will be determined using Simoa HD-1 Analyzer to explore whether the target protein levels changed before and after taVNS intervention and whether this change is correlated with consciousness (25).

### 2.13. Statistical analysis

Main statistical analyses will be performed at the individual and group levels (pre vs. post, treatment vs. sham) (26). To examine the differences between the groups, we shall employ the *t*-test or Mann–Whitney test for continuous variables and the Pearson Chi-squared or Fisher’s exact test for discrete values. If a statistically significant result appears, the inequality factors will be considered as potential confounding factors in the final analysis of effectiveness. To account for any potential external influences, linear models or linear regression will be implemented on continuous dependent variables and logistic regression models on categorical dependent variables. The Chi-square test or Fisher’s exact test will be employed to document and assess unfavorable events (26). We will test outcomes on behavior (diagnosis, CRS-R score) and brain data (MRI/EEG). The proportion of responders and the effect size of treatment (*r*, Cohen’s *d*) will be calculated. We will use *t*-tests, Chi-square tests, ANOVA (for normally distributed data), and Wilcoxon test (for non-ordinal data), and mixed-effect regression models, depending on the tested outcomes. EEG and MRI analysis will be evaluated using SPM12 software^1^ and EEGLAB^2^ implemented in MATLAB 9.5 (MathWorks, Natick, MA, United States), respectively. All other statistical analyses will be conducted in SPSS software (version 25). All data will be adjusted to account for multiple comparisons and considered statistically significant if the value of *p* is lower than 0.05.

## 3. Discussion

To date, no intervention strategy could provide evidence-based grade I evidence for patients with DoC patients (27). In 2018, we reported the effectiveness of peripheral sensory stimulation in patients with consciousness disorders (28). Vagus nerve stimulation is an important part of modern acupuncture and sensory stimulation. In our study, we observed behavioral improvement and brain activity in patients receiving sensory stimulation intervention (28). New investigations have demonstrated that stimulating the vagus nerve has an effect on DoC (11, 29–31). Despite this, the manner in which vagus nerve stimulation influences consciousness remains unclear.

Evidence has shown that there are three important structural bases for consciousness: the brainstem ascending reticular activation system (ARAS) (32), the central thalamus (33), and the posterior cingulate cortex (4). In addition, some brain networks are equally important for the recovery of consciousness, and understanding these brain neural correlates is critical for understanding the mechanism of action of taVNS. These neural networks include the default mode (DMN) (34), external parietal (ExN) (35), and salience network (SN) (36, 37). To better understand the mechanism of taVNS, the connection between these neural networks and brain structure must be clarified. Based on the central thalamus (lamina nucleus and related para-nucleus) and its main connection to the striatum and frontal cortex, the Mesocircuit model has been suggested as a potential explanation for the return of consciousness following severe head trauma (38, 39). Under this hypothesis, striatal dysfunction suppresses the central thalamic excitability state, which, in turn, results in a disruption of its excitatory cortical projection, and ultimately led to the DoC (4). The model states that the striatum suppresses the pallidus, which, in turn, suppresses the thalamus and pedunculopontine nucleus, and the striatum is sensitive to hypoxia, resulting in inhibition of the thalamus (4). It further disrupted the loop and exacerbated the inhibitory state (4). PET glucose metabolism showed significantly higher levels of the DoC medial pallidum compared to healthy subjects and lower levels in the central thalamus (4). Additionally, DoC patients showed a marked difference in the fractional anisotropy between the striatum and the pallidum in the left hemisphere compared to healthy controls (4). Moreover, the central loop model incorporates possible mechanisms of various intervention strategies that can explain the potential neuron conduction pathways to prevent DoC (8, 24, 40–45).

The goal of this protocol is to examine the consequences and operational aspects of taVNS in individuals suffering from DoC. The double-blind test design was implemented to corroborate the test outcomes. Additionally, an array of evaluation indexes including the CRS-R, EEG, MRI, and chemicals were employed to accurately and objectively gauge any alterations in the patient’s consciousness level (46). Ultimately, new medical data on the application of taVNS in DoC patients will be available.

### Trial status

The trial is anticipated to conclude by December 2023. Enrollment of participants commenced in April 2021.This protocol has a version number of ChiCTR2100045161.

## Ethics statement

The studies involving human participants were reviewed and approved by Hangzhou Normal University. Written informed consent to participate in this study was provided by the participants’ legal guardian/next of kin. Written informed consent was obtained from the individual(s), and minor(s)’ legal guardian/next of kin, for the publication of any potentially identifiable images or data included in this article.

## Author contributions

LC conceived and designed the study. LC and LS wrote the protocol and manuscript. FZ, LX, XL, and AW helped with the experiments. Y-SC and HD advised the experiments and revised the manuscript. All authors contributed to the article and approved the submitted version.

## Funding

This work was supported by the Department of Education of Zhejiang Province (Grant no. Y202147477), the Chen Xiaoying Education Fund of Hangzhou Normal University Qianjiang College (Grant no. 4065J2051911001) and National Natural Science Foundation of China (Grant no. 81920108023).

## Conflict of interest

The authors declare that the research was conducted in the absence of any commercial or financial relationships that could be construed as a potential conflict of interest.

## Publisher’s note

All claims expressed in this article are solely those of the authors and do not necessarily represent those of their affiliated organizations, or those of the publisher, the editors and the reviewers. Any product that may be evaluated in this article, or claim that may be made by its manufacturer, is not guaranteed or endorsed by the publisher.

## Footnotes

^1^http://www.fil.ion.ucl.ac.uk/spm/software/spm12/

^2^https://sccn.ucsd.edu/eeglab/ressources.php
