# Supplementary material for: Randomized trial of transcutaneous auricular vagus nerve stimulation on patients with disorders of consciousness: A study protocol
Source: Front Neurol. 2023 Apr 13;14:1116115. doi: 10.3389/fneur.2023.1116115 (PMC10133680; doi:10.3389/fneur.2023.1116115)
Supplement: Supplementary file 1 [file Data_Sheet_1.doc]

**EEG**

EEG signal acquisition will be performed using a 32-channel EEG device, with electrodes using a silver/silver chloride hybrid electrode, at the impedance between the skin and electrodes remained below 5 kΩ during data acquisition. During the data acquisition process, Participants will be required to always be behaviorally awake.

**MRI**

Before data collection, the name of the patient's immediate family members will be recorded through the sound acquisition software Goldwave6.35, and the music of interest will be recorded and edited and processed with the software. Stimulus presentation will be performed using a block design with 30s length and interval 30s, each containing 15 double syllables of SON-FV; During the data collection, the positioning image scanning will be first performed, and then the T1 weighted imaging sequence scanning will be used to obtain the structural image data. The data will be reconstructed through the multiplane sequence (magnetization-prepared, rapid acquisition gradient echo, MP-RAGE) for the acquisition, The parameter is set to: repetition time (TR)= 2300 ms; echo time (TE)=2.34 ms flip angle = 90°；slices = 160；field of view (FOV)= 256×256 mm^2^；matrix size = 256×256；voxel size = 1.0×1.0×1.0 mm^3^.Next, resting, calling / hand lift task-related functional images will be acquired using a gradient echo plane imaging sequence (echo-planar imaging, EPI), (echo-planar imaging, EPI). The parameter is set to: TR =2000 ms; TE= 30ms; flip angle= 90;slices = 35; FOV = 192x192 mm^2^; matrix size = 64x64; voxel size = 3.0x3.0x3.0 mm^3^; Finally, diffusion tensor imaging was performed (diffusion tensor imaging, DTI), using a single excitation EPI sequence, parameters Set to: TR = 800 ms; TE = 88 ms; FOV = 128128 mm^2^; matrix size = 128x128；direction = 64；total time = 12 min
